# New Appliances & Things Medical

**Published:** 1909-06-12

**Authors:** 


### NEW APPLIANCES & THINGS MEDICAL.

[We shall be glad to receive at our Office, 28 & 29 Southampton Street, Strand, London, W.C., from the manufacturers, specimens of all new preparations and appliances.]

### SYNOPTIC CHART OF CARDIAC EXAMINATION.

This chart, arranged by John D. Comrie, M.A., B.Sc., M.B., is intended for the use of both general practitioners and students. It consists of a cardboard sheath stamped with an outline of the chest and heart, inside of which there slides a sheet of cardboard. Upon the latter are printed the names of the various diseases that affect the heart, together with their physical signs. By the simple manipulation of two tapes, the different diseases are made to show in a space at one side of the chart, and, as each disease appears, its various signs automatically come into place at openings corresponding to the areas of auscultation, the borders of the heart, the situation of the pulse, etc.

Fuller information regarding the symptoms and physical signs of cardiac disease are given in a twelve-page pamphlet contained in an envelope upon the back of the chart; and this pamphlet comprises in brief most of what is correctly taught regarding the physical signs of disease in the organ in question. It is hoped the chart will prove valuable to the junior student of clinical medicine in the simplification of physical signs, which at first he is apt to find very bewildering; also to the senior student preparing for examination who desires a handy and concise memoriser; as well as to the general practitioner, who often wishes for a quick method of referring to the possible meanings of a given physical sign. The chart will be published shortly by Bale, Sons, and Danielsson, Ltd.

